# New approaches to organocatalysis based on C–H and C–X bonding for electrophilic substrate activation

**DOI:** 10.3762/bjoc.12.283

**Published:** 2016-12-23

**Authors:** Pavel Nagorny, Zhankui Sun

**Affiliations:** 1 Chemistry Department, University of Michigan, Ann Arbor, MI 48109, USA; 2 School of Pharmacy, Shanghai Jiao Tong University, No. 800 Dongchuan Rd., Shanghai 200240, P. R. China

**Keywords:** C–H hydrogen bond, counteranion activation, electrophile activation, halogen bond donor, hydrogen bond donor, organocatalysis

## Abstract

Hydrogen bond donor catalysis represents a rapidly growing subfield of organocatalysis. While traditional hydrogen bond donors containing N–H and O–H moieties have been effectively used for electrophile activation, activation based on other types of non-covalent interactions is less common. This mini review highlights recent progress in developing and exploring new organic catalysts for electrophile activation through the formation of C–H hydrogen bonds and C–X halogen bonds.

## Review

### Introduction

**Figure 1:**
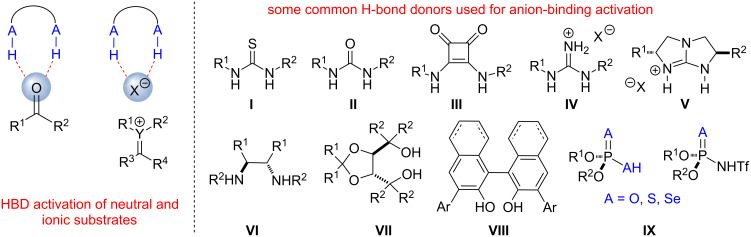
Electrophile Activation by Hydrogen Bond Donors [1–16].

Over the past century chemists have gathered great amounts of information about the factors governing enzymatic reactions. These studies have helped to realize the importance of non-covalent interactions within the receptor, and many subsequent efforts have been focused on adopting the knowledge learned from nature to the rational design of small molecule-based catalysts mimicking enzymatic function. A significant number of such efforts has been dedicated to designing new catalysts to enhance the electrophilicity of organic molecules through non-covalent interactions, and many important areas of organocatalysis have emerged from these efforts. The use of synthetic hydrogen bond donors for the activation of neutral or ionic electrophiles has been one of the major focuses of these research efforts in the past two decades (Figure 1) [1]. Many privileged hydrogen bond donor scaffolds capable of forming single or double hydrogen bonds with the substrate have been developed and explored as catalysts of numerous organic transformations [2–15]. Such catalysts (i.e., **I–IX**) [16] may contain one or several highly polarized A–H···A bonds, where A is oxygen or nitrogen and are often designed to mimic enzymatic reactions through the electrostatic substrate activation or stabilization of charged transition states or reaction intermediates. At the same time, recent studies highlight the importance of other types of non-covalent interactions such as the activation through the formation of C–H···A hydrogen bonds or halogen bonding (C–X···X or C–X···A interactions) in organocatalyst design. Such non-covalent interactions have been traditionally viewed as “weak” when compared to classical A–H···A hydrogen bonds. However, in some cases the term “weak” may be misleading as an increasing number of examples demonstrate the effectiveness of such interactions for organocatalyst design. While C–H···A hydrogen bonds have been invoked in biological processes, halogen bonding is not commonly observed in natural enzyme-catalyzed reactions. Therefore, application of these new interactions for small molecule activation allows expanding the repertoire of existing organic catalysts beyond what is found in biological systems.

This mini review highlights recent progress on organocatalysis that is based on C–H···A or halogen (C–X···X or C–X···A) bonds for substrate activation.

### C–H hydrogen bonds

**Figure 2:**
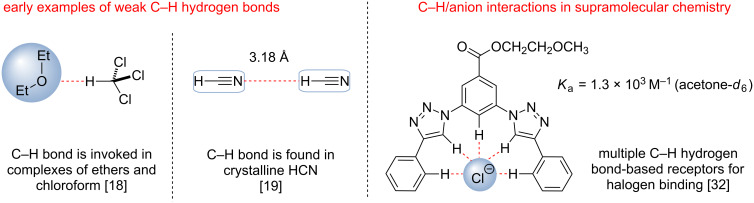
Early examples of C–H hydrogen bonds and their recent use in supramolecular chemistry [18–19,32–34].

It is well established that the C–H moiety can serve as a hydrogen bond donor and form hydrogen bonds with oxygen, nitrogen or halogens of neutral molecules or anions [17]. However, such interactions have been considered negligible in comparison to much stronger A–H···A hydrogen bonds (A = N, O, F). While Glasstone proposed the formation of a C–H hydrogen bond between chloroform and ethereal solvents in 1935 [18], and Lipscomb discovered hydrogen bonding in solid hydrogen cyanide in 1951 [19], until recently C–H hydrogen bonds have been mostly observed in the solid state (Figure 2). Recent studies in supramolecular chemistry have demonstrated that hydrogen bonds formed by C–H bonds are not necessarily “weak”, and in certain cases are almost as strong as more traditional A–H···A bonds [20]. The C–H hydrogen bonding between the substrate and the catalyst could be of great significance for transition state organization and energy, and is often invoked to rationalize the outcome of various transformations [21–27]. However, until recently C–H hydrogen bond-based interactions have not been employed in rational organic catalyst design, and more traditional A–H hydrogen bond donors such as **I–IX** (Figure 1) have been utilized to enhance the electrophilicity of organic molecules.

Recent spectroscopic and computational studies provided evidence that arenes might form strong hydrogen bonds between aryl C–H groups and anions (Cl^−^, NO_3_^−^, ClO_4_^−^, etc.) in the gas phase [28]. In addition, the introduction of an electron-withdrawing group into the aryl ring (i.e., NO_2_, CN, CF_3_, etc.) could significantly enhance this binding and result in stronger hydrogen bonds between the arene and the anion. Thus, the gas-phase binding energy of the nitrobenzene and chloride anion complex containing two C–H hydrogen bonds was estimated to be −16.8 kcal/mol whereas the corresponding binding energy for the H_2_O/Cl^−^ complex was determined to be −15.4 kcal/mol. Electron-deficient heterocyclic compounds such as 1,2,3-triazoles may also serve as strong C–H hydrogen bond donors. Substituted 1,2,3-triazoles possess a substantial dipole moment (≈4.5 D) almost aligned with the C5–H bond and the relatively high acidity of this position (p*K*_a(DMSO)_ = 27–28, for the 1*H*-tautomer). These heterocycles, which are easily available from 1,3-cycloaddition of alkynes and azides, can both form strong C–H bonds with hydrogen bond acceptors and also act as electron-withdrawing substituents when attached to other aromatic rings thus enhancing benzene’s ring C–H hydrogen bonding [29–31]. Recent studies by the Flood group and the Craig group suggested that receptors containing arylated 1,2,3-triazoles could form stable supramolecular complexes with anions [32–34]. The stability of such complexes correlated with the number of C–H bonds that could be formed by the receptor, and strong binding comparable to the more traditional X–H bonding based hydrogen bond donors was observed for the receptors forming 5–9 hydrogen bonds with halides (*K*_a_ ≈ 10^3^–10^4^ M^−1^ in acetone-*d*_6_). Not surprisingly, a higher number of hydrogen bonds with an anion correlated with the higher stability of the receptor/anion complex.

### 1,2,3-Triazole-based catalysts for the dearomatization of *N*-heteroarenes

**Scheme 1:**
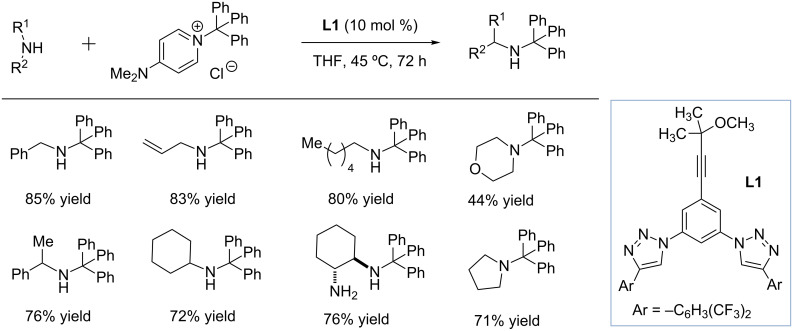
Design of 1,2,3-triazole-based catalysts for trityl group transfer through chloride anion binding by Mancheno and co-workers [35].

The Mancheno group recently explored triazole-based receptor **L1** as the organic catalysts for counterion activation (Scheme 1) [35]. Receptor **L1** capable of forming 5 hydrogen bonds was found to form a stable supramolecular complex with chloride in acetone (*K*_a_ = 458 M^−1^ in acetone-*d*_6_), and promoted efficient trityl group transfer from tritylated DMAP chloride to various primary and secondary amines. The authors propose that **L1** binding with chloride results in a more electrophilic tritylated DMAP cation, and the binding affinity of the catalyst was found to correlate with the *N*-alkylation rate.

**Scheme 2:**
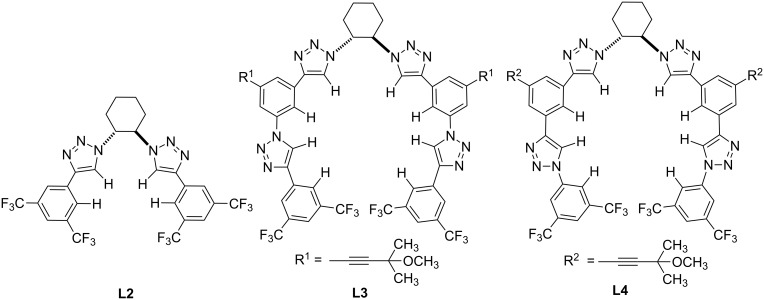
Examples of chiral triazole-based catalysts for anion activation designed by Mancheno and co-workers [36–39].

Following the aforementioned studies, the Mancheno group designed and synthesized various chiral triazole-based complexes such as **L2–L4** (Scheme 2) [36–39]. It was proposed that while these triazole derivatives are conformationally flexible, upon their binding to halogen anions these complexes adopt a reinforced chiral helical conformation. The resultant close chiral anion-pair complexes would then undergo a chiral counterion-controlled asymmetric reaction with a nucleophile. This proposal was validated experimentally, and a chloride-induced conformational switch to form a helical backbone was experimentally observed by circular dichroism (CD) during the titration of **L4** with TBAC [37].

**Scheme 3:**
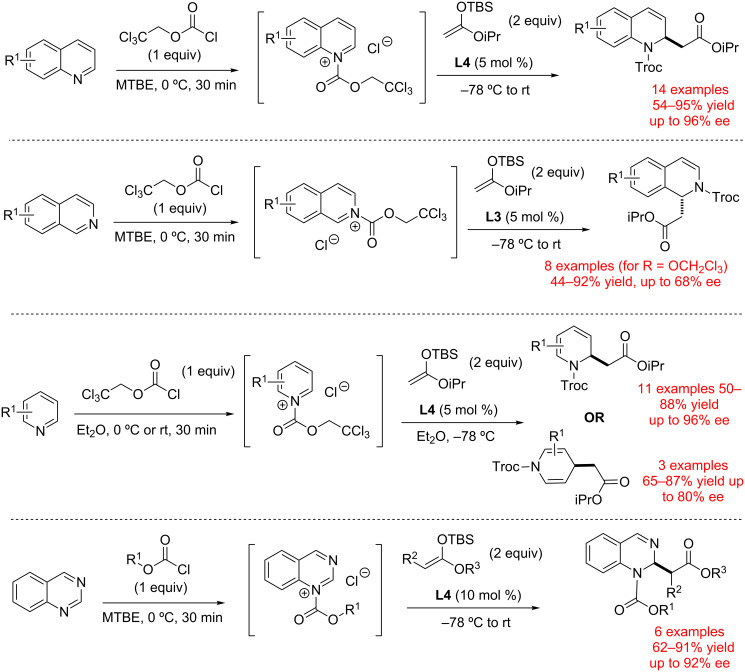
Application of chiral triazole-based catalysts **L3** and **L4** for counterion activation of pyridinium, quinolinium and isoquinolinium salts by Mancheno and co-workers [36–39].

Catalysts **L3** and **L4** were successfully applied to the asymmetric dearomatization of electron-deficient *N*-heteroarenes (Scheme 3). Various nitrogen-containing heterocycles such as pyridines [36], quinolines [38], isoquinolines [38], etc. were reacted with TrocCl to form the corresponding salts, and these generated in situ ion pairs were treated with silyl enol ethers in the presence of chiral catalysts **L2–L4** to form chiral addition products. High levels of chirality transfer were generally observed for various 6-membered nitrogen-containing heterocyclic substrates, and chiral products were obtained in good yields and selectivities. Remarkably, the performance of tetrakistriazoles was found to be comparable (or in some cases superior) to the well-established thiourea- and squaramide-based catalysts developed for similar transformations.

### Quaternary tetraalkylammonium and alkylpyridinium salts as C–H hydrogen bond donors

**Scheme 4:**
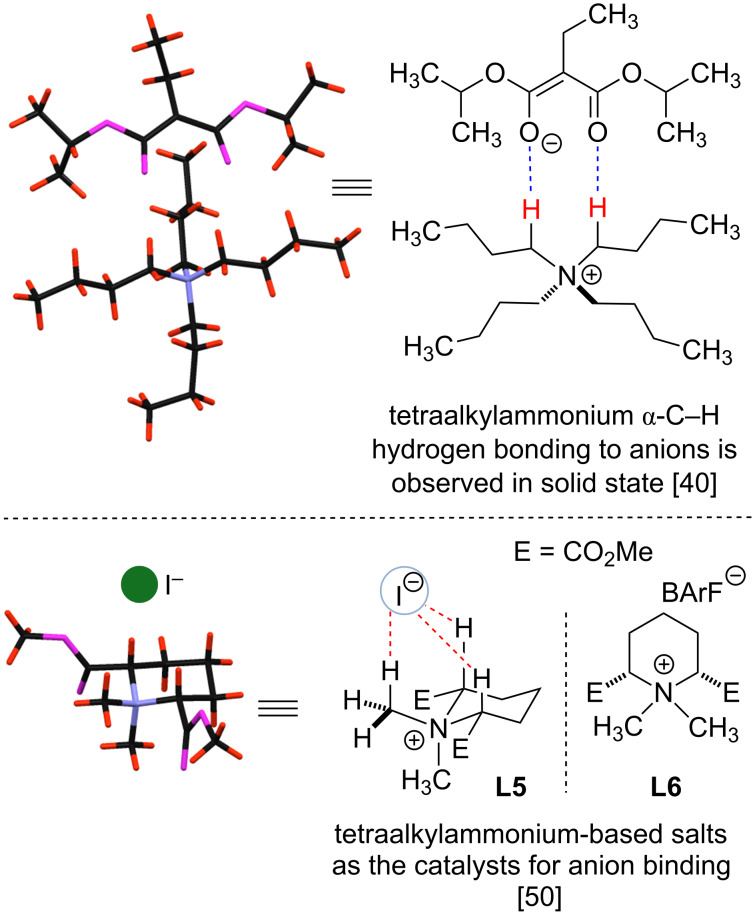
Ammonium salt anion binding via C–H hydrogen bonds in solid state [40–45,50–51].

While quaternary ammonium salts have extensively been used as phase transfer catalysts to activate ionic nucleophiles, recent studies suggest that these compounds can also serve as effective hydrogen bond donors. In 1993 Reetz and co-workers provided crystallographic evidence that the alpha C–H bonds of tetraalkylammonium salts are highly polarized, and can form multiple hydrogen bonds with enolates in the solid state (Scheme 4) [40–45]. The existence of alpha C–H hydrogen bonds has also been invoked in the computational studies rationalizing the outcome of various asymmetric phase-transfer reactions [46–49]. However, despite these developments until recently [50–51] the use of tetraalkylammonium salts as hydrogen bond donor catalysts has not been explored.

**Scheme 5:**
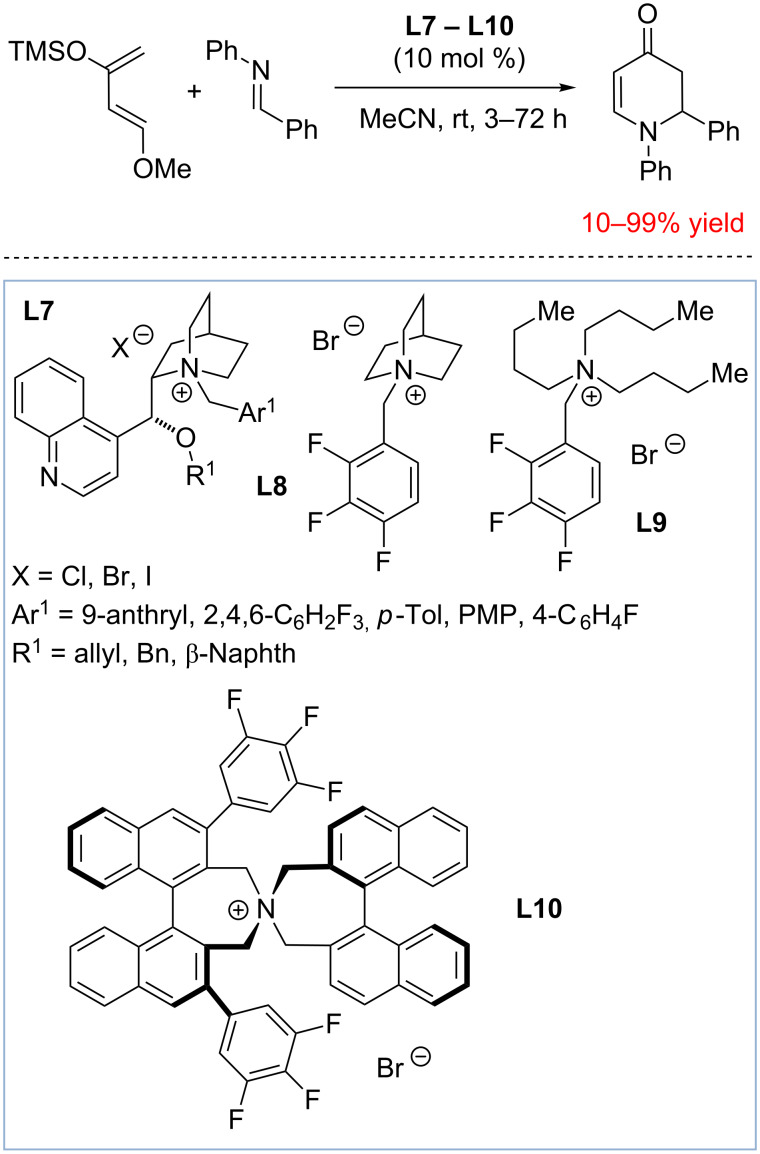
Early examples of ammonium salts being used for electrophilic activation of imines in aza-Diels–Alder reactions [52].

In 2011 the Park group investigated ammonium salts as catalysts for aza-Diels–Alder reactions of Danishefsky’s diene with imines (Scheme 5) [52]. A variety of ammonium salts (**L7–L10**) including chiral cinchonidine derivatives **L7** and catalyst **L10** were found to promote the reaction in low-to-good yields albeit with no enantioselectivity. Although it is perhaps one of the first examples of utilizing tetralkylammonium salts as hydrogen bond donor catalysts, the authors provided no mechanistic proposal for the catalytic activity of **L7–L10**, and the activation of the imine by the formation of a C–H···N hydrogen bond was proposed later by Maruoka and Shirakawa [50–51].

**Scheme 6:**
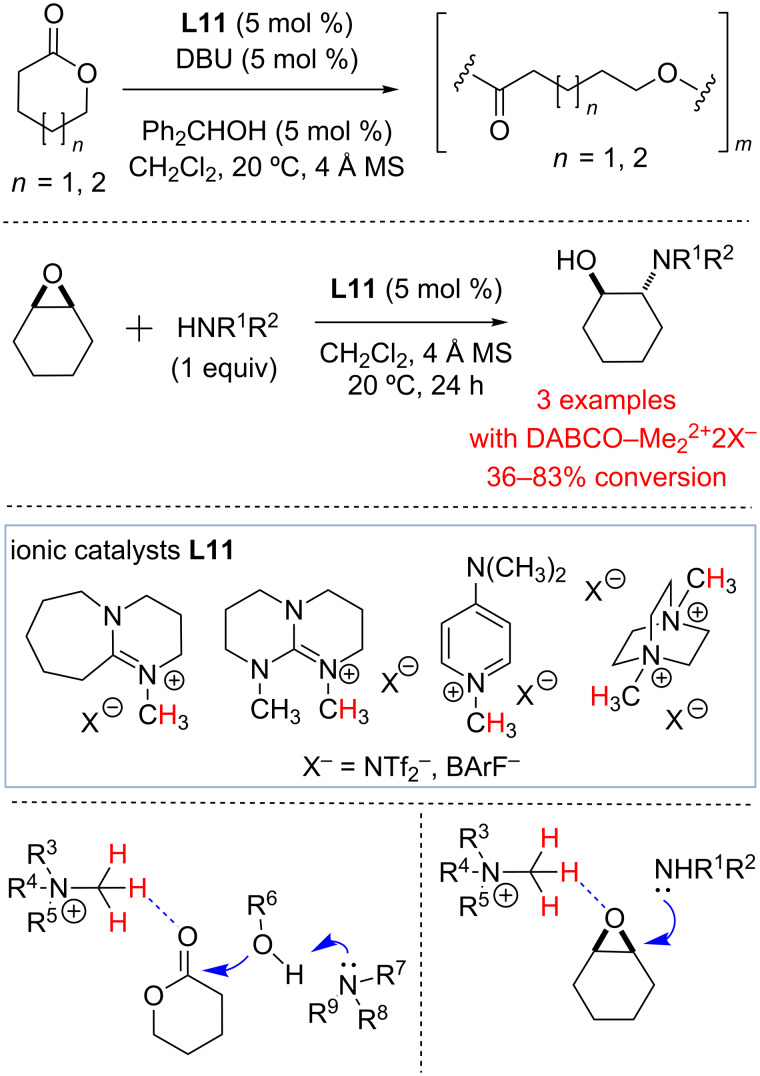
Ammonium salts as hydrogen bond-donor catalysts by Bibal and co-workers [53–54].

In 2013 Bibal and co-workers investigated the use of methylated amines, pyridines and guanidines (**L11**) as hydrogen bond-donor catalysts for the activation of cyclic esters toward ring-opening polymerization (ROP) [53]. Ionic catalysts **L11** (5 mol %) were successfully employed in combination with DBU and initiator (Ph_2_CHOH) to accomplish C=O activation and to promote the polymerization reactions (Scheme 6). Highly charged tetraalkylbisammonium salts (i.e., DABCO-Me_2_·2X) were found to be particularly active catalysts. Based on computational studies, the authors proposed that substrate activation is accomplished through a C–H hydrogen bond with cyclic ester carbonyls. The following study by the Bibal group described the use of catalysts **L11** as hydrogen bond donors for the activation of epoxides toward ring-opening aminolysis with amines (Scheme 6) [54]. Significant rate enhancement was observed under mild conditions with **L11**. The activity of catalysts **L11** was found to be comparable to the activity of common thiourea-based hydrogen bond donors, and double-charged catalyst DABCO-Me_2_·2X was found to be one of the most active catalysts. As before [53], the activity of ammonium salts **L11** was attributed to their ability to form a hydrogen bond with the oxygen of epoxide.

**Scheme 7:**
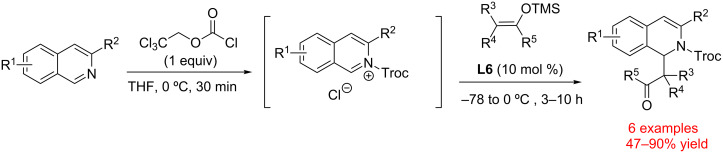
Tetraalkylammonium catalyst (**L6**)-catalyzed dearomatization of isoquinolinium salts [50].

In 2015 Shirakawa and Maruoka demonstrated that ammonium salts **L5** and **L6** could serve as effective catalysts for isoquinolinium and pyridinium salt Mannich reactions (Scheme 7) [50]. Catalysts **L5** and **L6** were selected due to their conformational rigidity that results in a better alignment of alpha C–H groups, relatively high polarization of the alpha C–H bonds due to the presence of electron-withdrawing ester functionalities, and ease of preparation (1–2 steps from commercially available piperidine). Interestingly, the authors assessed the strength of C–H hydrogen bonds to the iodide anion in **L5** relative to the *N*-methylpiperidinium iodide salt by measuring the distances between the alpha-hydrogen atom and iodide in the solid state. Thus, the C–H···I hydrogen bonds for **L5** were found to be 0.2–0.4 Å shorter than for *N*-methylpiperidinium iodide.

**Scheme 8:**
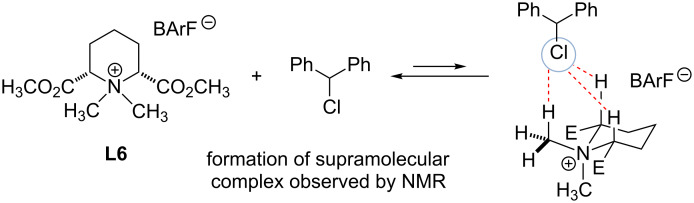
Tetraalkylammonium catalyst **L6** complexation to halogen-containing substrates [51].

Both **L5** and **L6** were found to promote the Mannich reaction between *N*-Troc-isoquinolinium chlorides and silyl enol ethers. Catalyst **L6** with non-coordinating BArF^−^ counterion was found to have a significantly higher activity than **L5** with iodide counterion, probably, due to the competitive binding with iodide. Both **L5** and **L6** were inhibited by the addition of tetrabutylammonium chloride, which reinforces the proposal that these salts act as HBDs. Interestingly, NMR titration studies revealed that **L6** could form a supramolecular complex not only with a chloride anion, but also with chlorine atoms covalently bonded to a benzylic carbon (Scheme 8).

**Scheme 9:**
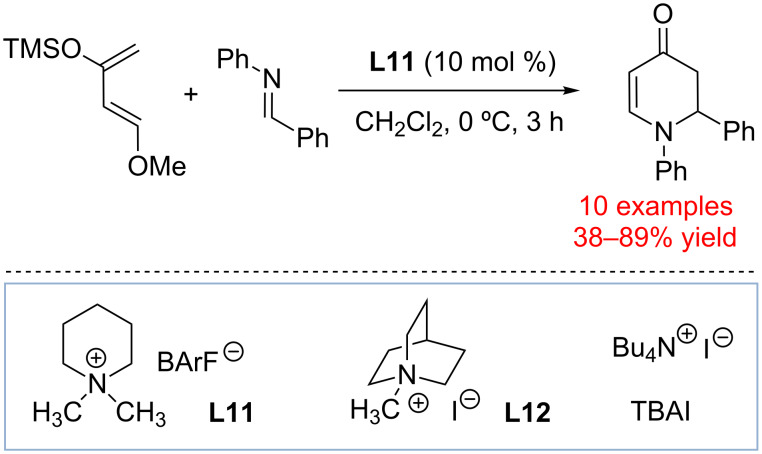
Tetraalkylammonium-catalyzed aza-Diels–Alder reaction by Maruoka and co-workers [52].

In 2016 Maruoka and Shirakawa followed up the aforementioned studies by demonstrating that tetraalkylammonium salts **L5**, **L6**, **L11** and **L12** as well as TBAI could activate imines toward aza-Diels–Alder reaction with Danishefsky’s diene (Scheme 9) [51]. Catalyst **L11** was selected as the catalyst of choice due to its simplicity and activity. Based on the ^1^H NMR studies and X-ray crystallographic analysis the authors concluded that tetralkylammonium salts act as hydrogen bond donors through the formation of a C–H···N hydrogen bond.

**Scheme 10:**
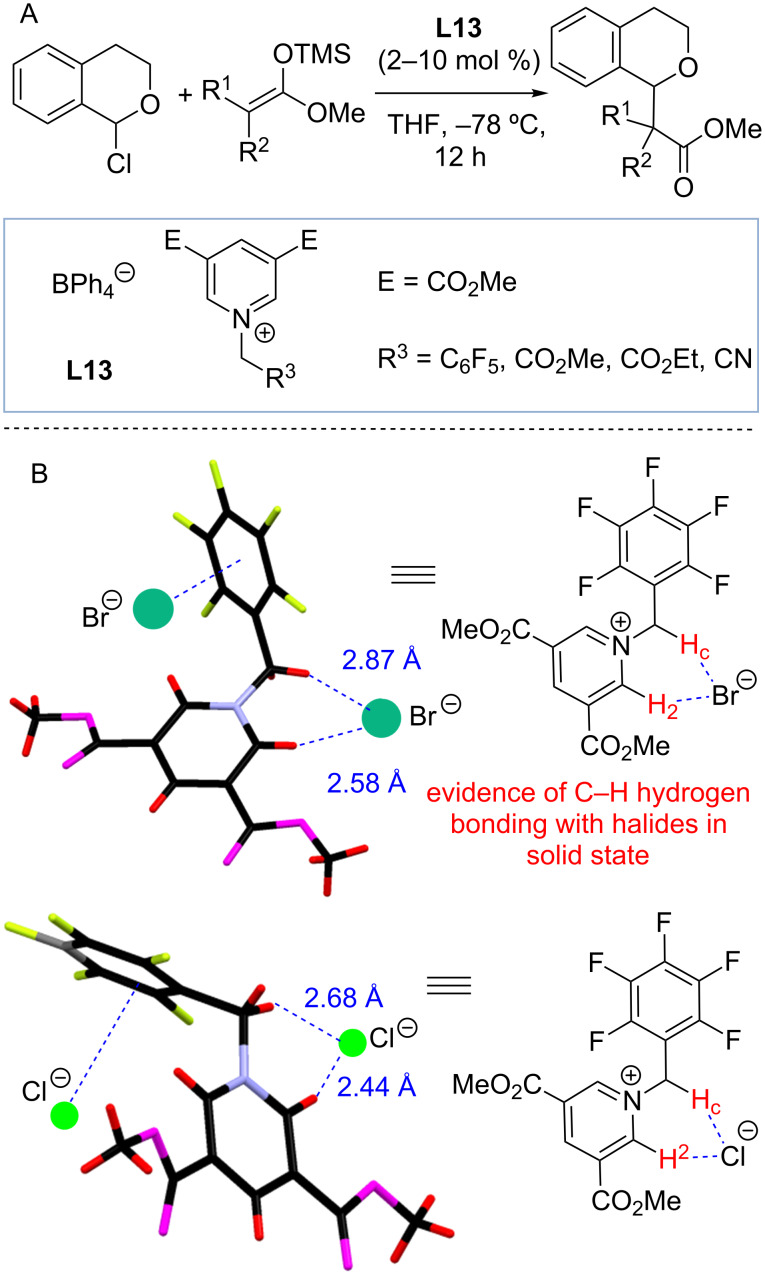
(A) Alkylpyridinium catalysts **L13**-catalyzed reaction of 1-isochroman and silyl ketene acetals by Berkessel and co-workers. (B) Evidence of **L13** C–H···X^–^ hydrogen bonding in solid state [55].

In 2014, Berkessel and co-workers reported the use of *N*-alkylated 3,5-di(carbomethoxy)pyridinium ions **L13** to catalyze the reaction between 1-chloroisochroman and silyl ketene acetals (Scheme 10A). Catalyst **L13** with R^3^ = C_6_F_5_ was found to be particularly active, and was found to efficiently form the product at 2 mol % loading without significant erosion in yield or reaction time [55]. Interestingly, the ability of catalysts **L13** to catalyze the reaction was attributed to 1-chloroisochroman activation through Coulombic interactions coupled with anion–π bonding. Thus, **L13** was proposed to promote ionization of 1-chloroisochroman followed by anion exchange. The resultant oxocarbenium/tetraphenylborate ion pair undergoes a nucleophilic attack by silyl ketene acetal, which is followed by scavenging the trimethylsilyl cation with a chloride anion to result in chlorotrimethylsilane and the product. Mechanistic studies were conducted to establish that **L13** forms weak 1:1 complexes with chloride and bromide anions. Thus, ^1^H NMR titration studies of **L13** with R^2^ = C_6_F_5_ demonstrated the formation of the corresponding chloride complex with 1:1 stoichiometry and *K*_a_ ≈ 200 M^−1^.

In light of the recent studies by Shirakawa and Maruoka [50–51], we propose that catalysts **L13** could act not only through Coulombic interactions, but also as hydrogen bond donors. While various factors including Coulombic interactions between the pyridinium (or ammonium) salt and the chloride undoubtedly play an important role in promoting substrate ionization and chloride complexation, the provided X-ray data are consistent with **L13** acting as hydrogen bond donors (Scheme 10B). The published X-ray data for the chloride and bromide salts of **L13** with R^2^ = C_6_F_5_ indeed provide evidence of anion–π bonding with the C_6_F_5_ group. In addition, we also noted that there is evidence for two hydrogen bonds formed between the halide or bromide anion and C–H_2_/C–H_c_ bonds of another cation **L13**.

### Mixed N–H/C–H hydrogen bond donors as organocatalysts

**Scheme 11:**
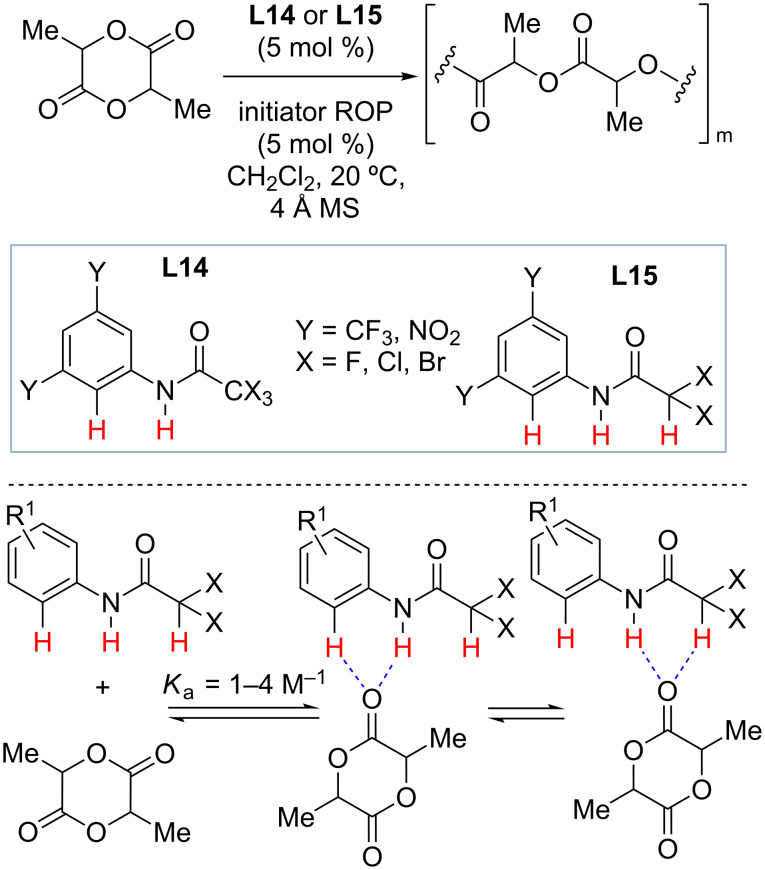
Mixed N–H/C–H two hydrogen bond donors **L14** and **L15** as organocatalysts for ROP of lactide by Bibal and co-workers [58].

The involvement of the ortho C–H bond in the binding event with Lewis-basic sites was proposed by Etter in the late 1980s and later demonstrated by Schreiner in a detailed study of hydrogen-bonding thiourea organocatalysts containing a 3,5-bis(trifluoromethyl)phenyl group as the privileged motif [56–58]. A recent example of utilizing such interactions in catalysis was demonstrated by Bibal and co-workers [58]. In this study, Bibal and co-workers explored the use of α-halogenated acetanilides **L14** and **L15** as hydrogen-bonding organocatalysts that activate the carbonyl functionality of lactide and thus enhance their reactivity toward ROP (Scheme 11). In addition to their ability to form more conventional N–H hydrogen bonds, **L14** and **L15** were proposed to form additional C–H hydrogen bonds between arene or α-halogenated acetyl groups and the carbonyl of lactide. X-ray crystallographic analysis and molecular modeling provided the evidence of such interactions in solid state, and the titration studies established weak binding (*K*_a_ ≈ 1–4 M^−1^) between **L14** or **L15** and lactide in solution. The α-dichloro and α-dibromoacetanilides **L14** containing electron-deficient aromatic groups (i.e., *m,m*’-NO_2_ substitution on phenyl ring) afforded the most active catalysts with the strongest N–H···O···H–CX_2_ interactions.

### Halogen bonds as alternatives to hydrogen bonds

**Scheme 12:**
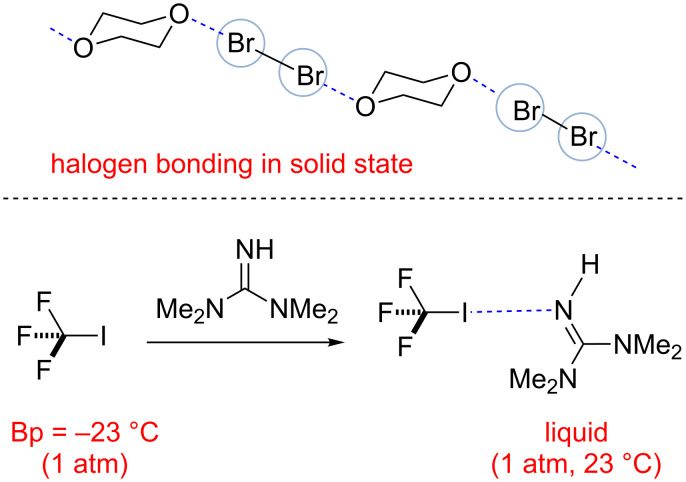
Examples of stable complexes based on halogen bonding [68–69].

The ability of halogens and halogenated organic compounds to form stable complexes with nucleophiles has been known for more than two centuries [59–61]. One of the earliest examples of such complexes were the adducts formed by the reaction of iodine and amylose or ammonia described by Colin in 1814 [62]. While the stoichiometry of such complexes was not established at the time, Guthrie in 1863 [63] and Norris in 1896 [64] were able to generate complexes formed between molecular halogens (I_2_, Br_2_, and Cl_2_) and ammonia or methylamines and characterized them. Numerous studies attempting to elucidate the nature of halogen complexes have emerged since then; however, the structural features of these interactions were unclear until the work of Mulliken who proposed the formation of donor–acceptor complexes [65–66] and Odd Hassel who conducted crystallographic studies of bromine complexed with 1,4-dioxane in 1970 [67]. The evidence of the actual bonding were found in these complexes as the O−Br distance in the crystal was about 2.71 Å (Scheme 12), which is 20% smaller than the sum of the van der Waals radii of oxygen and bromine (3.35 Å) while the angle between the O−Br and Br−Br bond was found to be ≈180°.

Since these reports, numerous other examples of halogen bonding have been uncovered, and halogen bonding has become of great importance to the fields of chemistry and materials science. Not only halogens, but also neutral halogen-containing organic molecules were found to form stable adducts with neutral and charged Lewis bases, and to account for this IUPAC provided the following recommendation “A halogen bond occurs when there is evidence of a net attractive interaction between an electrophilic region associated with a halogen atom in a molecular entity and a nucleophilic region in another, or the same, molecular entity” [68]. Such complexes may have substantially different properties from the uncomplexed organohalides, which is well exemplified by the at room temperature liquid complex of gaseous iodotrifluoromethane (BP = −23 ºC) and tetramethylguanidine containing a halogen (I···N) bond [70].

**Scheme 13:**
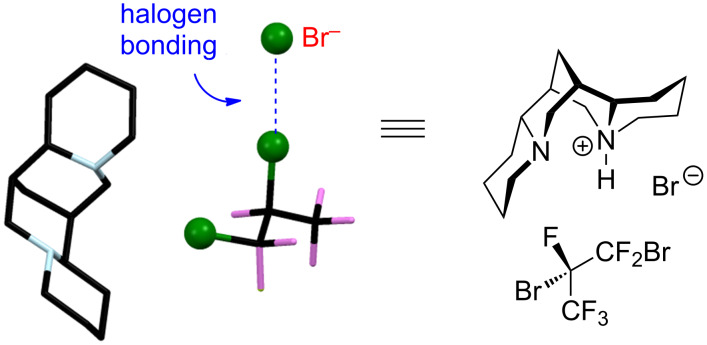
Interaction between (−)-sparteine hydrobromide and (*S*)-1,2-dibromohexafluoropropane in the cocrystal through halogen bonds [69].

When chiral organohalides form halogen bonds with chiral acceptors, diastereomeric complexes may be formed. Thus, in 1999, Resnati reported the resolution of racemic 1,2-dibromohexafluoropropane through halogen-bonded supramolecular helices (Scheme 13) [69]. When (−)-sparteine hydrobromide in chloroform was treated with racemic 1,2-dibromohexafluoropropane, a yellow co-crystal was isolated. The structure of the co-crystal was confirmed by single-crystal X-ray diffraction, and it showed that the co-crystal was made up from one molecule of (−)-sparteine hydrobromide and one molecule of (*S*)-1,2-dibromohexafluoropropane. The Br^−^···Br distance (Scheme 13) is about 3.3 Å, which is approximately 20% shorter than the sum of the van der Waals radii. The angle between Br^−^···Br–C is about 175º. The strong Br^−^···Br–C halogen bonds are robust enough to drive the self-assembly and are critical for the resolution. This example clearly demonstrate the great potential of halogen bonds as tools for asymmetric catalysis.

### Early uses of halogen bond donors in catalysis and organocatalysis

**Scheme 14:**
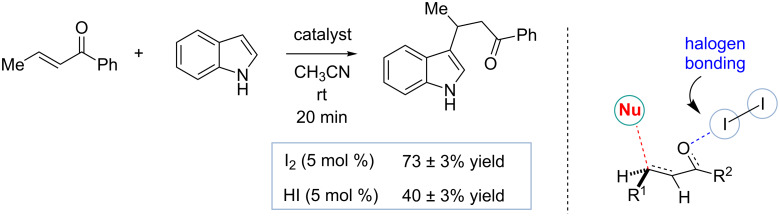
Iodine-catalyzed reactions that are computationally proposed to proceed through halogen bond to carbonyl [80].

Like hydrogen bonds, halogen bonds possess important features such as strength and directionality that might make these interactions of a great value to the field of organocatalysis [71–73]. Molecular iodine has been used for many decades as a mild catalyst or promoter of various organic transformations such as conjugate addition, imine formation or aldolate dehydration reactions [74–79]. Interestingly, such reaction mechanisms are not well understood, and the formation of trace quantities of hydroiodic acid rather than the direct substrate activation by molecular iodine has been frequently invoked to rationalize the outcome of these studies. Recently, Breugst and co-workers have re-evaluated the molecular iodine-catalyzed conjugate addition to α,β-unsaturated carbonyls or nitrostyrenes (Scheme 14) [80]. Based on their computational studies, they proposed that iodine activates the enone moiety by forming a halogen bond with the carbonyl and thus forming a more reactive complex with the LUMO. These results are further backed up by control experiments demonstrating that catalytic quantities of hydroiodic acid were less effective in promoting this reaction than molecular iodine, and hidden Brønsted acid catalysis is unlikely to be operational in these studies.

**Scheme 15:**
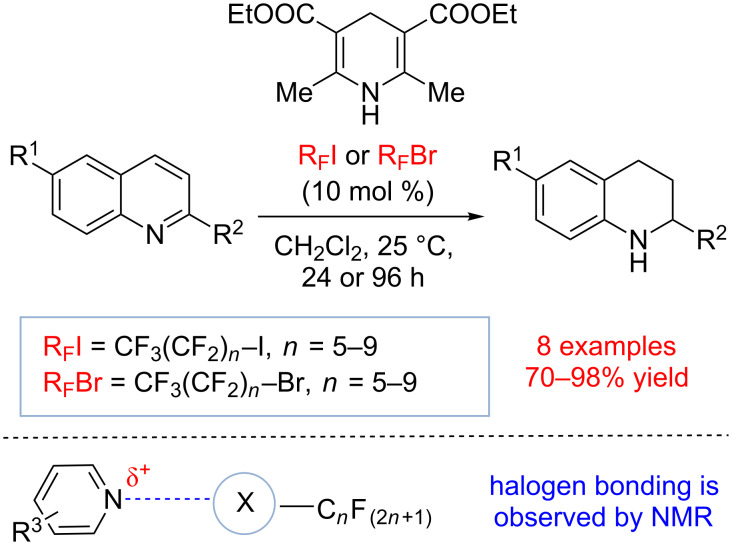
Transfer hydrogenation of phenylquinolines catalyzed by haloperfluoroalkanes by Bolm and co-workers [81].

In 2008 the Bolm group explored the use of halogen bond donors in organocatalysis. They discovered that perfluorinated alkyl halides could activate 2-substituted quinolines toward reduction by Hantzsch ester (Scheme 15) [81]. These studies explored various C_6_ to C_10_ perfluorinated bromides and iodides. It was discovered that organic iodides were more reactive than the corresponding bromides and that the product yield increased with increasing lengths of the perfluorinated carbon chain. Thus, CF_3_(CF_2_)_9_I was found to be the most effective at 10 mol % loading and the product could be isolated in 98% yield after 24 h. Remarkably, when the catalyst loading was reduced to 1 mol %, the product could still be obtained in 69% yield after 96 h. Although the hidden acid catalysis due to the trace amounts of HI was not ruled out, the proposed *N*-halogen bond formation between quinolone and perfluoroiododecane during the catalytic process was supported by ^13^C and ^19^F NMR studies.

### Halogen bond donor organocatalysis based on aryl halides

**Scheme 16:**
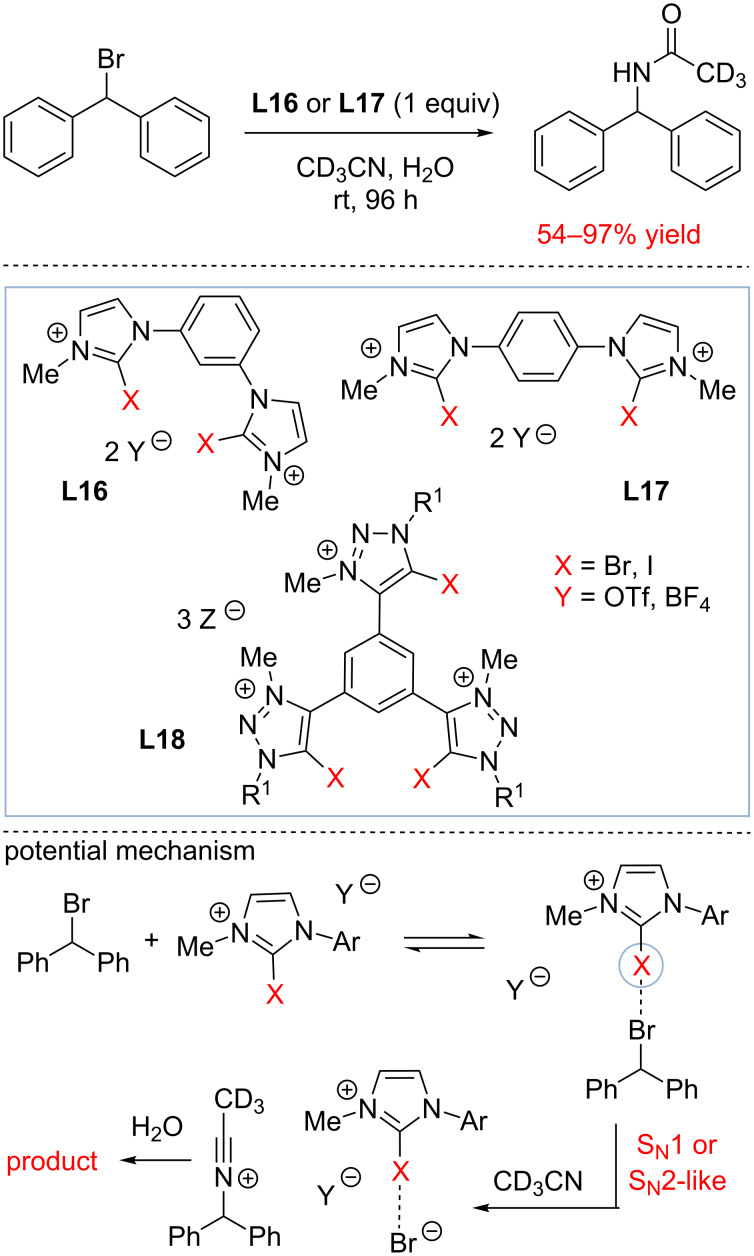
Halogen bond activation of benzhydryl bromides by Huber and co-workers [82].

The pioneering study of Bolm has attracted significant attention and a number of important studies have emerged since then. In particular, the Huber group has contributed to the development of a new design of organocatalysts based on aryl iodides as halogen bond donors [73,82–88]. These catalysts were found to act as organic Ag^+^ surrogates and activate an ionizable substrate by halide anion scavenging. Thus, in 2011, Stefan M. Huber and his colleagues investigated the activation of a C–Br bond by novel halogen-bond donors **L16** and **L17** [82]. The authors synthesized compounds **L16** and **L17** as well as some other halogen bond donors and tested their ability to promote the Ritter reaction of (bromomethylene)dibenzene (Scheme 16). The stoichiometric amounts of **L16** and **L17** were found to promote the formation of a benzhydryl acetamide product in good-to-excellent yields. The control experiments with deiodinated **L16** and **L17** confirmed the importance of halogen bonding for this transformation. A strong counterion effect was observed in these studies, and the BF_4_^−^ salt of **L16** were found to be significantly more reactive than the corresponding TfO^−^ salts. Interestingly, **L16** that can potentially form two halogen bonds with the same bromine atom were found to be marginally less active promoters in comparison to compounds **L17**. Finally, the control experiments ruled out hidden acid catalysis due to the formation of trace quantities of HBr through hydrolysis of the substrate.

**Scheme 17:**
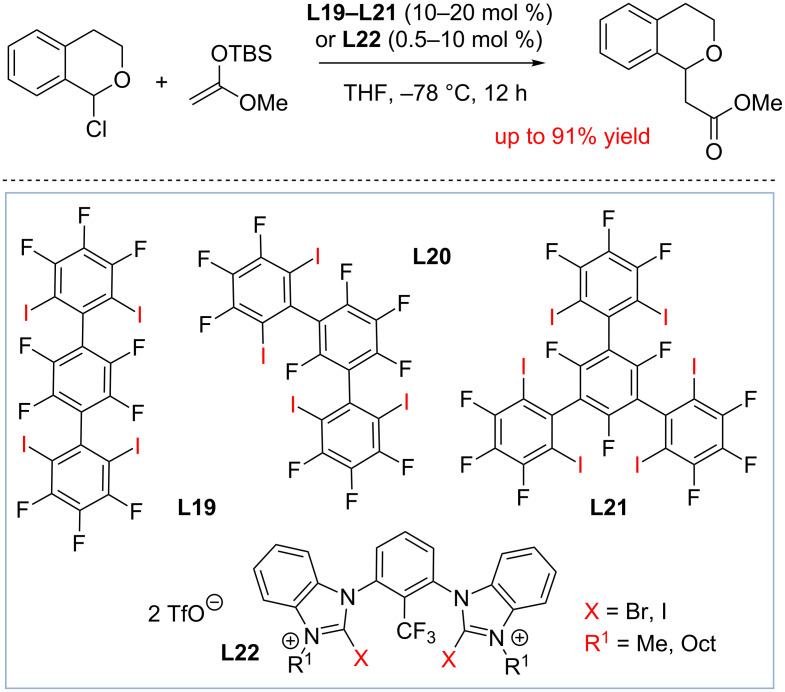
Halogen bond-donor-catalyzed addition to oxocarbenium ions by Huber and co-workers [89].

To further enhance the reactivity of the halogen bond donors, Huber and colleagues later designed 5-iodo-1,2,3-triazolium-based multidentate salts **L18** [83–84]. Triazolium salts **L18** were found to be particularly good in promoting the formation of benzhydryl acetamide (95% after 96 h). Due to the formation of the strong inhibitor, hydrobromic acid that acts as the source of bromide anion as the reaction progresses, the aforementioned studies required a stoichiometric amount of halogen bond donors. However, catalytic halogen scavenging with halogen bond donors is also possible if the products are not inhibiting the catalyst. One of such transformations explored by the Huber group is the addition of ketene silyl acetals to 1-chloroisochroman (Scheme 17) [85,87]. The chloride anion produced in this reaction will eventually form chlorotrialkylsilane, which does not inhibit the catalyst. As a result, catalytic amounts of neutral HBDs **L19–L21** were found to promote this transformation at 10–20 mol % catalyst loading. Unlike the Ritter reaction study summarized in Scheme 16, the activity of the catalyst was found to directly correlate with the stability of supramolecular complex, and **L21** capable of forming multiple halogen bonds at the same time was found to be the most active catalyst. The binding properties of **L19–L21** toward halides in the solid state, in solution, and in the (calculated) gas phase were further investigated by Huber [89].

Following the aforementioned studies, the Huber group has designed structurally rigid electron-deficient cationic catalysts **L22** based on the bis(halobenzimidazolium) scaffold [87]. These catalysts were found to be particularly active, and the catalyst with X = I, and R = Oct promoted the reaction between 1-chloroisochroman and silyl enol ether at 0.5 mol % catalyst loadings (70% yield, 6 h). A good correlation was observed between the catalytic activity and halogen affinity, and the *K*_a_ of **L22** with X = I, R = Oct with bromide anion was determined to be 3.5 × 10^6^ M^−1^ (CH_3_CN). In addition to the aforementioned studies, recent results by Huber and Codée indicate that not only 1-chloroisochroman, but also more complex substrates such as 2,3,4,6-tetra-*O*-benzylglucosyl chloride could undergo halogen bond-donor-catalyzed solvolysis [86].

**Scheme 18:**
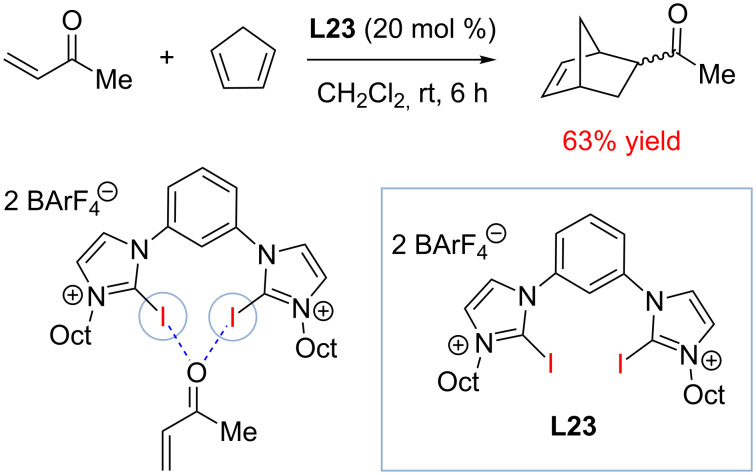
Halogen bond-donor activation of α,β-unsaturated carbonyl compounds in the [2 + 4] cycloaddition reaction of MVK and cyclopentadiene [88].

Further studies by the Huber group and others suggest that halogen bond donors based on the bis(halobenzimidazolium) scaffold could promote other types of reactions that are typically observed with hydrogen bond donors such as thioureas [88]. Thus catalyst **L23** was found to catalyze the reaction of methyl vinyl ketone (MVK) and 1,3-cyclopentadiene (Scheme 18).

Catalyst **L23** was proposed to form two halogen bonds with the carbonyl group of MVK and thus activate it by lowering the energy of its LUMO. Remarkably, the non-coordinating counterion BArF_4_^−^ was required for **L23** to act as the catalyst with the less coordinating TfO^−^ anion did not accelerate the cycloaddition.

**Scheme 19:**
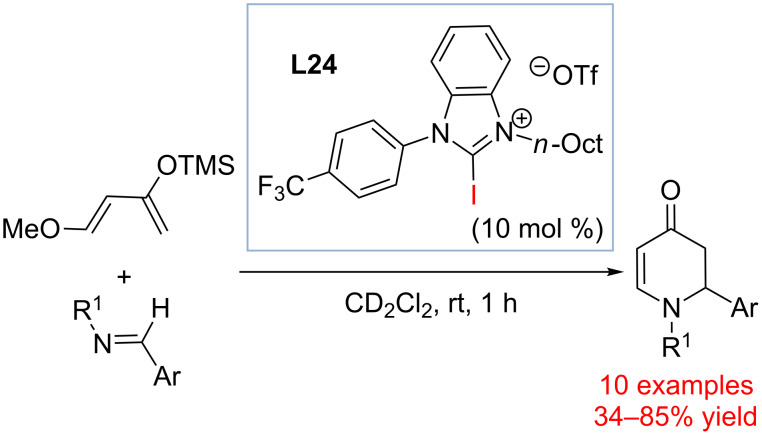
Halogen bond donor activation of imines in the [2 + 4] cycloaddition reaction of imine and Danishefsky’s diene [90].

In 2015, Takeda, Minakata and co-workers demonstrated that 2-iodoimidazolium salt **L24** could serve as an efficient catalyst of the aza-Diels−Alder reaction of aldimines and Danishefsky diene (Scheme 19) [90].

Other organohalides such as perfluoroiodooctaine or perfluoroiodobenzene were initially explored as the catalysts; however, no product formation was observed. The following evaluation of iodoimidazoles and iodoiimidazolium salts helped identifying **L24** as the catalyst of choice (yield 85%). When **L24** lacking the iodide activating site was used, no product was formed. The halogen bond nature of the reaction was further confirmed by adding an acid scavanger (K_2_CO_3_) that does not inhibit the reaction and by including *n*-Bu_4_NCl, which inhibited the reaction. The authors also did titration experiments and ^1^H NMR studies. These results strongly supported that halogen bonding was the key interaction for catalyst action.

**Scheme 20:**
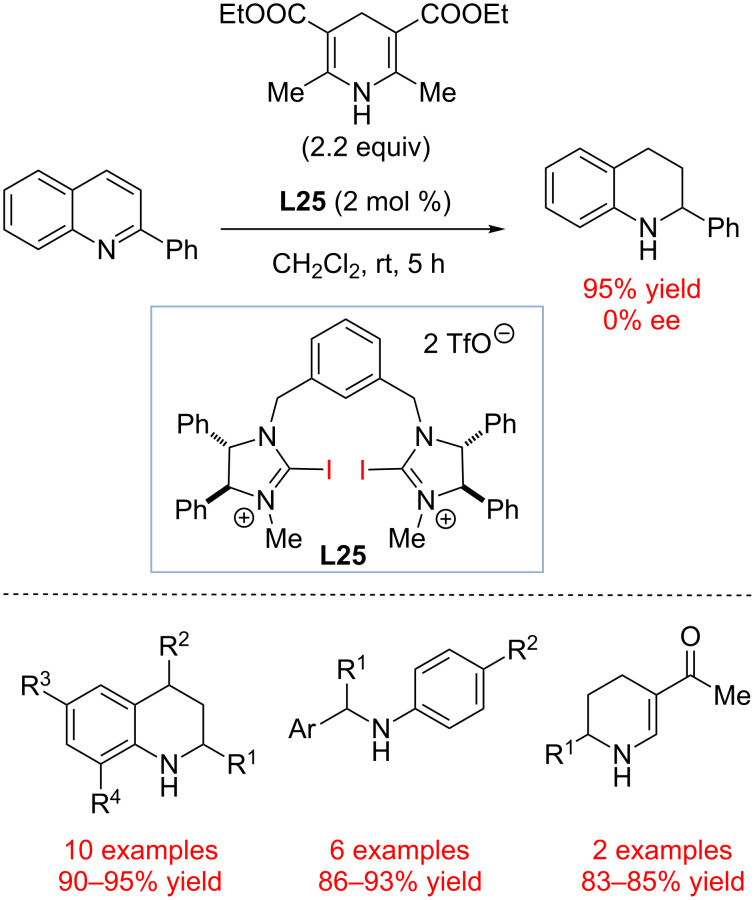
Transfer hydrogenation catalyzed by a chiral halogen bond donor by Tan and co-workers [91].

In 2014, the Tan group revisited the original study by Bolm and co-workers and re-investigated halogen bond induced hydrogen transfer to C=N bonds (Scheme 20) [91]. Various charged and uncharged electron-deficient iodoarenes were tested as potential catalysts, and chiral catalyst **L25** was identified as the catalyst of choice. Although no chirality transfer was observed during the reduction of 2-phenylquinoline, **L25** was found to be a very active catalyst promoting transfer hydrogenation of a C=N group containing heterocycles and imines with significantly greater reaction times than CF_3_(CF_2_)_9_I originally published by the Bolm group. As before, ^1^H NMR studies and calorimetric titration were used to validate the proposed halogen bond-based activation of substrates.

**Scheme 21:**
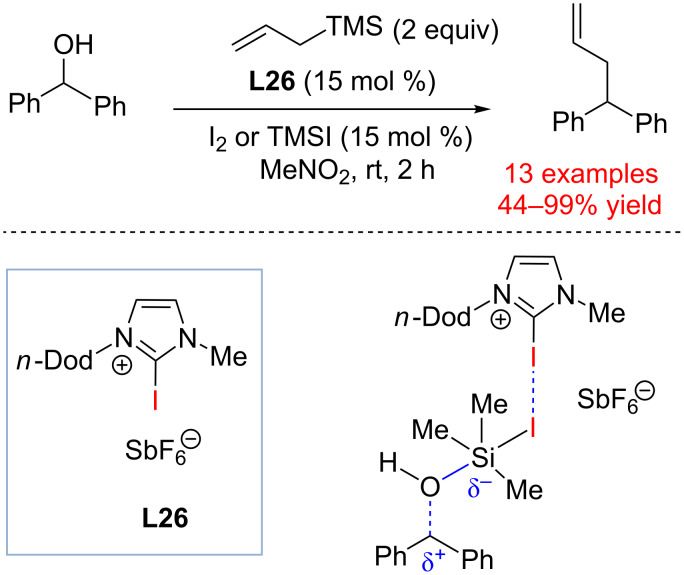
Allylation of benzylic alcohols by Takemoto and co-workers [92].

Halogen bond-donor catalysis can be relevant to the processes in which a halogen-containing acceptor is formed in transient quantities. Thus, in 2015, Takemoto and co-workers described a halogen bond donor-catalyzed dehydroxylative coupling reaction of benzyl alcohols and allyltrimethylsilane (Scheme 21) [92].

This transformation requires catalytic quantities of the halogenating agent (I_2_ or TMSI, 15 mol %). These additives may undergo activation by **L26** to result in more Lewis acidic species that ionize benzylic alcohols. Alternatively, addition of I_2_ or TMSI may accomplish in situ transformation of benzylic alcohols to benzyl iodides. These intermediates underwent coordination to halogen bond donor catalyst **L26** to provide an electrophilic complex equivalent to a benzyl cation. This substrate was trapped with allyltrimethylsilane to provide the corresponding product. It was found that catalyst **L26** required a hexafluoroantimonate (SbF_6_^−^) counterion and polar MeNO_2_ as the solvent of choice. Other nucleophiles such as trimethyl(phenylethynyl)silane, trimethylsilanecarbonitrile and (cyclohex-1-en-1-yloxy)trimethylsilane could be used as substrates albeit with somewhat lower yield. Importantly, catalyst **L26** was found not to bind iodotrimethylsilane, which is the condition essential for achieving turnover in this reaction.

### Organocatalysis based on halogen bond donors with *N*-halogenated moiety

**Scheme 22:**
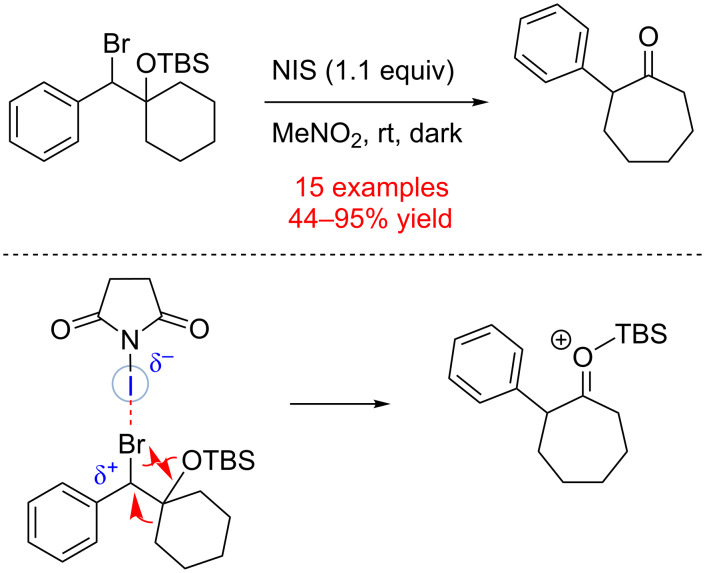
NIS induced semipinacol rearrangement via C–X bond cleavage [93].

A variety of *N*-halogenated organic molecules has been developed and utilized for halo-functionalization of olefins. Considering the electrophilic nature of a halogen in such molecules, these compounds could serve as effective halogen bond donors. In 2014, Takemoto and co-workers reported that electrophilic *N*-iodinated compounds could induce a semipinacol rearrangement (Scheme 22) [93]. Thus, the benzyl bromide moiety of substrates was activated upon treatment with *N*-iodosuccinimide (NIS) in nitromethane. The resultant carbocation-like species presumably underwent a 1,2-alkyl shift to provide a silylated oxocarbenium ion. The following silyl cation trapping with a bromide anion resulted in 2-phenylcycloheptanone. When a chiral substrate (59% ee) was treated with NIS, a product with significantly lower ee (11%) was observed. These results suggest that the reaction might proceed mainly in a stepwise S_N_1-like manner, via a benzylic carbocation intermediate.

Despite the fact that several variants of chiral halogen bond-donor catalysts have been synthesized, to the best of our knowledge no successful asymmetric catalytic transformation based on the direct substrate activation with a chiral halogen bond donor has been reported. Therefore, this represents a fruitful area for further developments.

## Conclusion

While the N–H or O–H-based hydrogen bond donors have traditionally dominated the field of organocatalysis, numerous recent examples highlight the importance of other types of non-covalent interactions for electrophilic substrate activation. The use of C–H and C–X bonds with halogens or electron-rich heteroatoms has been particularly useful in new organocatalyst design. These interactions (and C–H hydrogen bonds in particular) have been traditionally ascribed as “weak”; however, this term could be rather misleading as both C–H hydrogen bonds as well as halogen bonds could be similar in strength to hydrogen bonds. Not surprisingly, some of the examples highlighted in this review demonstrate that the catalysts based on such interactions could match or even outperform the existing O–H/N–H hydrogen bond donors such as thioureas. Several different variants of C–H hydrogen bond donors have been developed; however, the most successful designs have involved the use of 1,2,3-triazole, ammonium and pyridinium based catalysts. While the asymmetric transformations catalyzed by chiral 1,2,3-triazoles are now well precedented, the feasibility of asymmetric transformations promoted by other types of chiral C–H hydrogen bond donors is yet to be demonstrated. Similarly, compared with the N–H and O–H hydrogen bond catalysis, asymmetric halogen bond-donor catalysis is still underdeveloped and the possibility of utilizing chiral halogen bond donors for the asymmetric transformations is yet to be demonstrated. This is somewhat surprising, given the fact that a number of different scaffolds for halogen bond donors containing C–X bonds (including the chiral variants) have been developed. Considering that the use of organic halogen bond donors could offer some significant advantages, it is reasonable to anticipate the emergence of asymmetric halogen bond-donor catalysis in the near future.
